# Polo-like kinase (PLK) 5, a new member of the PLK family, serves as a biomarker to indicate anabatic tumor burden and poor prognosis for resectable non-small cell lung cancer

**DOI:** 10.3389/fsurg.2022.964044

**Published:** 2023-01-06

**Authors:** Kaichao Wang, Shaohui Shen, Liyuan Dong, Qinmo Fang, Xinlei Hou, Xueliang Shi

**Affiliations:** ^1^ Department of Cardiothoracic Surgery, Daqing Oilfield General Hospital, Daqing, China; ^2^ Department of Cardiothoracic Surgery, Longnan Hospital, Daqing, China; ^3^ Department of Gynecology, Daqing Oilfield General Hospital, Daqing, China

**Keywords:** polo-like kinase 5, non-small cell lung cancer, clinical characteristics, disease-free survival, overall survival

## Abstract

**Objective:**

A review argues that polo-like kinase 5 (PLK5) may be linked to unfavorable prognosis in non-small cell lung cancer (NSCLC) patients, which contradicts the discoveries from The Human Protein Atlas database (derived from TCGA analysis). This study intended to comprehensively confirm the association of PLK5 with clinical characteristics and prognosis in NSCLC patients.

**Methods:**

This two-center, retrospective, cohort study enrolled 210 NSCLC patients receiving surgical resection. PLK5 protein and mRNA were detected by immunohistochemistry and RT-qPCR in tumor and nontumor tissues. Moreover, RNA FPKM data for 994 lung cancer patients were obtained from The Human Protein Atlas database.

**Results:**

PLK5 protein was decreased in tumor tissue compared to nontumor tissue (*P *< 0.001). Additionally, decreased PLK5 protein was linked with increased pathological grade (*P *= 0.002), lymph node metastasis presence (*P *= 0.001), elevated tumor-node-metastasis (TNM) stage (*P *= 0.003), and abnormal cancer antigen 125 (CA125) (*P *= 0.002). Meanwhile, low PLK5 protein was correlated with shortened disease-free survival (DFS) (*P *= 0.007) and overall survival (OS) (*P *= 0.038); further multivariable Cox regression analysis revealed that low PLK5 protein independently predicted unfavorable DFS (hazard ratio = 0.573, *P *= 0.022). PLK5 mRNA was reduced in tumor tissue compared with nontumor tissue (*P *< 0.001); its decline was linked with enhanced pathological grade (*P *= 0.034), climbed TNM stage (*P *= 0.032), and abnormal CA125 (*P *= 0.002). Furthermore, low PLK5 mRNA was correlated with unfavorable DFS (*P *= 0.046). The Human Protein Atlas database also disclosed the link between low PLK5 mRNA and worse OS (*P *= 0.046).

**Conclusion:**

A PLK5 decrement reflects anabatic tumor burden and poor prognosis in NSCLC patients.

## Introduction

Non-small cell lung cancer (NSCLC) is the primary category of lung cancer and has a high incidence and mortality risk worldwide (1–3). Currently, the fundamental treatment options include surgical resection, chemotherapy, targeted therapy, immunotherapy, radiotherapy, etc. (4–7). Among these, for NSCLC patients with tumor node metastasis (TNM) stage I–III, the mainstream treatments include surgical resection, neoadjuvant therapy followed by surgical resection, or surgical resection combined with adjuvant therapy (8–10). However, due to the risk of recurrence in tumors, the prognosis of NSCLC patients is still not optimistic (11, 12). Therefore, it is fundamental to discover candidate biomarkers to better realize risk stratification and improve the management of NSCLC patients.

The polo-like kinase (PLK) family (e.g., PLK1-5) plays a fundamental role in the pathogenesis and progression of tumors (13, 14); however, limited studies have explored the value of PLK5 in cancer. A study reveals that PLK5 deletion is related to the metastasis of clear cell renal cell carcinoma (15). In addition, a review demonstrates the potential antitumor role of PLK5 in tumorigenicity (14). Regarding the clinical role of PLK5 in NSCLC, a secondary study based on the Gene Expression Profiling Interactive Analysis (GEPIA) database shows that elevated PLK5 is linked to a worse prognosis in NSCLC patients (16). However, according to The Human Protein Atlas data [derived from The Cancer Genome Atlas (TCGA)], increased PLK5 is linked to a better prognosis in NSCLC patients (Supplementary Figure S1), which conflicts with the study mentioned above (16). As a result, the clinical role of PLK5 in NSCLC remains elusive.

Accordingly, the present study applied immunohistochemistry (IHC) and reverse transcription-quantitative polymerase chain reaction (RT-qPCR) to detect PLK5 protein and mRNA expression, aiming to confirm its relationship with clinical characteristics and prognosis in NSCLC patients.

## Methods

### Subjects

In this two-center cohort study, two hundred and ten patients with primary NSCLC who underwent surgical resection between March 2016 and February 2020 were retrospectively reviewed. Patients who met the following criteria were eligible for inclusion: (a) diagnosis of NSCLC per European Society for Medical Oncology (ESMO) clinical recommendations (17); (b) over 18 years old; (c) received surgical resection; (d) had available formalin-fixed paraffin-embedded (FFPE) specimens of tumor tissues for study use; and (e) had available clinical information. Patients with a prior history of other primary carcinomas or malignant hematologic diseases were excluded. This study was approved by the Ethics committee of Daqing Oilfield General Hospital (ethical approval number: KS1952). Written or verbal tape-recording informed consent was collected from each patient or the family.

### Collection of data

The clinical features of NSCLC patients were obtained for analysis. Additionally, standardized follow-up data of NSCLC patients were also obtained, and the final follow-up date was March 1, 2022. Disease-free survival (DFS) and overall survival (OS) were imputed. Patients who lost follow-up were recorded as survivors and censored at the last contact date.

### Collection of specimens

A total of 210 FFPE specimens of tumor tissues and 30 samples of nontumor tissues were collected to detect PLK5 protein expression for exploration analysis. In addition, a total of 80 fresh-frozen samples of tumor tissues and 30 specimens of nontumor tissues were collected to quantify PLK5 mRNA expression for validation analysis.

### IHC

PLK5 protein expression was evaluated by IHC assay, as reported in a previous study (18, 19). Rabbit polyclonal anti-PLK5 antibody (Cat., PA5-72987, dilution 1:200, Thermo Fisher, USA) was used as the primary antibody, and goat anti-rabbit IgG H + L (Cat., 65-6120, dilution 1:2000, Thermo Fisher, USA) was used as the secondary antibody. After staining, IHC results were assessed using a light microscope, which was the product (ranging from 0 to 12) of the staining intensity score (ranging from 0 to 3) and staining density score (ranging from 0 to 4). For analysis, tumor PLK5 protein expression was classified based on the IHC score into low expression (IHC score ≤3) and high expression (IHC >3).

### RT-qPCR

PLK5 mRNA expression was assessed by RT-qPCR assay. In brief, total RNA was extracted by TRIzol™ Reagent (Thermo Fisher Scientific, USA). Reverse transcription was performed using the iScript™ cDNA Synthesis Kit (Bio-Rad, USA). Subsequently, qPCR was carried out *via* a QuantiNova SYBR Green PCR Kit (Qiagen, Germany). Finally, PLK5 mRNA expression was calculated by the 2^−ΔΔCt^ method. The primers were referenced from previous literature (15). For analysis, tumor PLK5 mRNA expression was classified based on the median value (0.324) into low expression (≤0.324) and high expression (>0.324).

### Collection of PLK5 RNA fragments of kilobase million (FPKM) data

Nine hundred ninety-four lung cancer patients' RNA FPKM data were obtained from The Human Protein Atlas (derived from TCGA analysis, available at https://www.proteinatlas.org/ENSG00000185988-PLK5/pathology/lung+cancer) to further verify the correlation of PLK5 expression with OS.

### Statistics

Data analyses were carried out using SPSS 26.0 (IBM Corp., USA). Graphs were made using GraphPad Prism 7.02 (GraphPad Software Inc., USA). PLK5 expression between two kinds of tissues was examined using a paired-samples t test or Wilcoxon signed-rank test. PLK5 expression in patients with different characteristics was compared using Student's t test, one-way analysis of variance (ANOVA), Wilcoxon rank-sum test, or Kruskal–Wallis H rank-sum test, as appropriate. The correlation of PLK5 expression with disease characteristics was assessed using Spearman's rank correlation test. The correlation of PLK5 expression with DFS and OS was presented using the Kaplan–Meier curve and analyzed using the log-rank test. Prognostic analysis was completed using forward-stepwise multivariate Cox proportional hazard regression analysis with all factors included. *P* < 0.05 was considered significant.

## Results

### Study flow

Totally, 256 NSCLC patients were screened, while 46 patients were excluded, including 12 patients who refused to participate, 29 patients who did not have available clinical information, and 5 patients who did not have available FFPE specimens. Subsequently, 210 NSCLC patients were analyzed. Firstly, the clinical data was collected, containing clinical features and follow-up data. Secondly, the specimen was collected, including 210 FFPE specimens of tumor tissues, 30 FFPE specimens of nontumor tissues, 80 fresh-frozen specimens of tumor tissues, and 30 fresh-frozen specimens of nontumor tissues. Thirdly, the FPKM data was collected, including 994 lung cancer patients' RNA FPKM data (Supplementary Figure S2).

### Clinical features

The mean age of NSCLC patients was 61.2 ± 11.8 years, with 59 (28.1%) females and 151 (71.9%) males. Regarding underlying diseases, 64 (30.5%), 40 (19.0%), and 25 (11.9%) patients had hypertension, hyperlipidemia, and diabetes, respectively. There were 109 (51.9%), 93 (44.3%), and 8 (3.8%) patients with adenocarcinoma (ADC), squamous cell carcinoma (SCC), and others, respectively. Concerning pathological grade, there were 34 (16.2%), 109 (51.9%), and 67 (31.9%) patients in grade 1, grade 2, and grade 3, respectively. Additionally, 95 (45.2%) patients had a tumor size ≤5 cm, and 115 (54.8%) patients had a tumor size >5 cm. In addition, 83 (39.5%) patients had lymph node (LYN) metastasis. Regarding TNM stage, there were 34 (16.2%), 93 (44.3%), and 83 (39.5%) patients in stage I, stage II, and stage III, respectively. Regarding the Eastern Cooperative Oncology Group performance status (ECOG PS) score, there were 141 (67.1%) patients with a score of 0 and 69 (32.9%) patients with a score of 1. Finally, the median (interquartile range (IQR)) values of carcinoembryonic antigen (CEA) and cancer antigen 125 (CA125) were 5.3 (2.5–30.7) ng/mL and 32.4 (13.1–79.8) U/mL, respectively (Table 1).

**Table 1:** Clinical features.

Items	NSCLC patients (*N* = 210)
Age (years), mean ± SD	61.2 ± 11.8
Gender, no. (%)	
Female	59 (28.1)
Male	151 (71.9)
Smoker, no. (%)	103 (49.0)
Drinker, no. (%)	75 (35.7)
Hypertension, no. (%)	64 (30.5)
Hyperlipidemia, no. (%)	40 (19.0)
Diabetes, no. (%)	25 (11.9)
Subtype, no. (%)	
ADC	109 (51.9)
SCC	93 (44.3)
Others	8 (3.8)
Pathological grade, no. (%)	
Grade 1	34 (16.2)
Grade 2	109 (51.9)
Grade 3	67 (31.9)
Tumor size, no. (%)	
≤5 cm	95 (45.2)
>5 cm	115 (54.8)
LYN metastasis, no. (%)	83 (39.5)
TNM stage, no. (%)	
I	34 (16.2)
II	93 (44.3)
III	83 (39.5)
ECOG PS score, no. (%)	
0	141 (67.1)
1	69 (32.9)
CEA (ng/ml), median (IQR)	5.3 (2.5–30.7)
CA125 (U/ml), median (IQR)	32.4 (13.1–79.8)

NSCLC, non-small cell lung cancer; SD, standard deviation; ADC, adenocarcinoma; SCC, squamous cell carcinoma; LYN, lymph node; TNM, tumor-node-metastasis; ECOG PS, Eastern Cooperative Oncology Group performance status; CEA, carcinoembryonic antigen; IQR, interquartile range; CA125, cancer antigen 125.

### Neoadjuvant and adjuvant therapy information

In total, 80 (38.1%) NSCLC patients received neoadjuvant chemotherapy, of which 47 (22.4%), 15 (7.1%), 14 (6.7%), and 4 (1.9%) patients received navelbine + cisplatin (NP), docetaxel + cisplatin or carboplatin (DP), gemcitabine + cisplatin or carboplatin (GP), and taxol + cisplatin or carboplatin (TP) regimens, respectively, while 13 (6.2%) patients received neoadjuvant immune checkpoint inhibitors (ICIs). Additionally, a total of 167 (79.5%) NSCLC patients underwent adjuvant chemotherapy, of which 96 (45.7%), 27 (12.9%), 24 (11.4%), and 20 (9.5%) patients underwent NP, DP, GP, and TP regimens, respectively, while 19 (9.0%) patients received adjuvant ICI (Table 2).

**Table 2:** Neoadjuvant and adjuvant therapy.

Items	NSCLC patients (*N* = 210)
Neoadjuvant chemotherapy, no. (%)	80 (38.1)
NP regimen	47 (22.4)
DP regimen	15 (7.1)
GP regimen	14 (6.7)
TP regimen	4 (1.9)
Neoadjuvant ICI, no. (%)	13 (6.2)
Adjuvant chemotherapy, no. (%)	167 (79.5)
NP regimen	96 (45.7)
DP regimen	27 (12.9)
GP regimen	24 (11.4)
TP regimen	20 (9.5)
Adjuvant ICI, No. (%)	19 (9.0)

NSCLC, non-small cell lung cancer; NP, navelbine + cisplatin; DP, docetaxel + cisplatin or carboplatin; GP, gemcitabine + cisplatin or carboplatin; TP, taxol + cisplatin or carboplatin; ICI, immune checkpoint inhibitor.

### PLK5 protein expression

**Figure 1:**
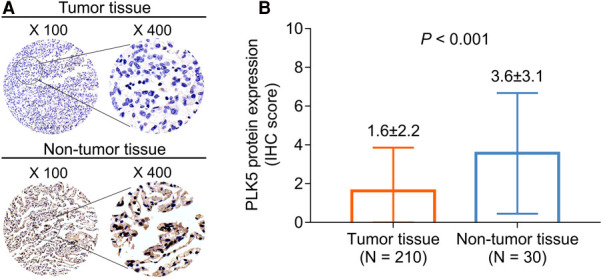
Comparison of PLK5 protein expression between tumor and nontumor tissues in NSCLC patients. PLK5 protein samples in tumor and nontumor tissues (**A**); PLK5 protein expression was reduced in tumor tissue in contrast with nontumor tissue in NSCLC patients (**B**).

IHC examples of tumor and nontumor tissues are presented (Figure 1A). PLK5 protein expression was decreased in tumor tissue {IHC score [mean ± standard deviation (SD)]: 1.6 ± 2.2} compared with nontumor tissue [IHC score (mean ± SD): 3.6 ± 3.1] in NSCLC patients (*P *< 0.001) (Figure 1B).

### Relationship of tumor PLK5 protein expression with disease characteristics

Decreased tumor PLK5 protein expression was related to increased pathological grade (*P *= 0.002), the presence of LYN metastasis (*P *= 0.001), elevated TNM stage (*P *= 0.003), and CA125 > 35 U/ml (*P *= 0.002). At the same time, no association was found between PLK5 protein expression and other disease features, including subtype, tumor size, ECOG PS score, and CEA in NSCLC patients (all *P *> 0.05) (Table 3).

**Table 3:** Correlation of tumor PLK5 protein expression with disease characteristics.

Items	Tumor PLK5 protein expression (IHC score), mean ± SD	*P* value
Subtype		0.484
ADC	1.5 ± 2.1	
SCC	1.8 ± 2.4	
Others	1.8 ± 1.8	
Pathological grade		0.002
Grade 1	2.8 ± 3.0	
Grade 2	1.6 ± 2.2	
Grade 3	1.0 ± 1.6	
Tumor size		0.188
≤5 cm	1.9 ± 2.2	
>5 cm	1.4 ± 2.2	
LYN metastasis		0.001
No	2.0 ± 2.3	
Yes	1.0 ± 2.0	
TNM stage		0.003
I	2.2 ± 2.2	
II	1.9 ± 2.5	
III	1.1 ± 1.9	
ECOG PS score		0.314
0	1.7 ± 2.2	
1	1.4 ± 2.3	
CEA		0.441
≤5 ng/ml	1.8 ± 2.3	
>5 ng/ml	1.5 ± 2.1	
CA125		0.002
≤35 U/ml	2.1 ± 2.6	
>35 U/ml	1.1 ± 1.7	

PLK5, polo-like kinase 5; IHC, immunohistochemistry; SD, standard deviation; ADC, adenocarcinoma; SCC, squamous cell carcinoma; LYN, lymph node; TNM, tumor-node-metastasis; ECOG PS, Eastern Cooperative Oncology Group performance status; CEA, carcinoembryonic antigen; CA125, cancer antigen 125.

### Linkage of tumor PLK5 protein expression with DFS and os

**Figure 2:**
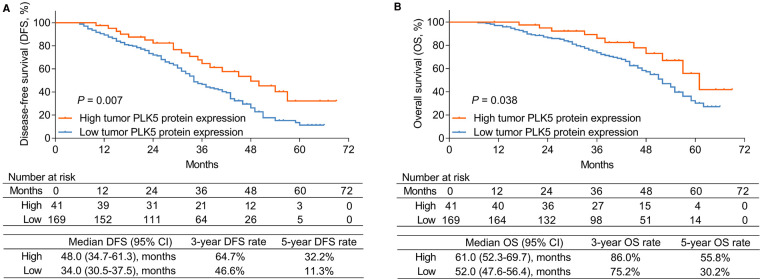
Relationship between PLK5 protein expression and survival profile in NSCLC patients. Linkage of PLK5 protein expression with DFS (**A**) and OS (**B**) in NSCLC patients.

High tumor PLK5 protein expression was related to prolonged DFS in NSCLC patients (*P *= 0.007). Meanwhile, the median [95% confidence interval (CI)] DFS of patients with high tumor PLK5 protein expression was 48.0 (34.7–61.3) months, with a 3-year DFS rate of 64.7% and a 5-year DFS rate of 32.2%; for patients with low tumor PLK5 protein expression, the median (95% CI) DFS was 34.0 (30.5–37.5) months, with a 3-year DFS rate of 46.6% and a 5-year DFS rate of 11.3% in NSCLC patients (Figure 2A).

Concurrently, high tumor PLK5 protein expression correlated with prolonged OS in NSCLC patients (*P *= 0.038). The median (95% CI) OS of patients with high tumor PLK5 protein expression was 61.0 (52.3–69.7) months, with a 3-year OS rate of 86.0% and a 5-year OS rate of 55.8%; the median (95% CI) OS of patients with low tumor PLK5 protein expression was 52.0 (47.6–56.4) months, with a 3-year OS rate of 75.2% and a 5-year OS rate of 30.2% in NSCLC patients (Figure 2B).

### Factors related to DFS and os

According to the multivariable Cox regression analysis for DFS, tumor PLK5 protein expression (high vs. low) [hazard ratio (HR) = 0.573, *P *= 0.022] was independently related to prolonged DFS, whereas higher pathological grade (HR = 1.344, *P *= 0.024) and ECOG PS score (1 vs. 0) (HR = 2.185, *P *< 0.001) were both independently linked with shorter DFS. Multivariable Cox regression analysis for OS showed that higher pathological grade (HR = 1.604, *P *= 0.004), tumor size (>5 cm vs. ≤5 cm) (HR = 2.544, *P *< 0.001), and ECOG PS score (1 vs. 0) (HR = 2.074, *P *= 0.002) were all independently associated with poor OS in NSCLC patients (Table 4).

**Table 4:** Factors related to DFS and OS by cox's proportional hazards regression analysis.

Items	*P* value	HR	95% CI	
			Lower	Upper
**Multivariable Cox's regression analysis for DFS**				
Tumor PLK5 protein (high vs. low)	0.022	0.573	0.355	0.924
Higher pathological grade	0.024	1.344	1.040	1.737
ECOG PS score (1 vs. 0)	<0.001	2.185	1.538	3.104
**Multivariable Cox's regression analysis for OS**				
Higher pathological grade	0.004	1.604	1.163	2.213
Tumor size (>5 cm vs. ≤5 cm)	<0.001	2.544	1.660	3.898
ECOG PS score (1 vs. 0)	0.002	2.074	1.295	3.322

DFS, disease-free survival; OS, overall survival; HR, hazard ratio; CI, confidence interval; PLK5, polo-like kinase 5; ECOG PS, Eastern Cooperative Oncology Group performance status.

### PLK5 mRNA expression

**Figure 3:**
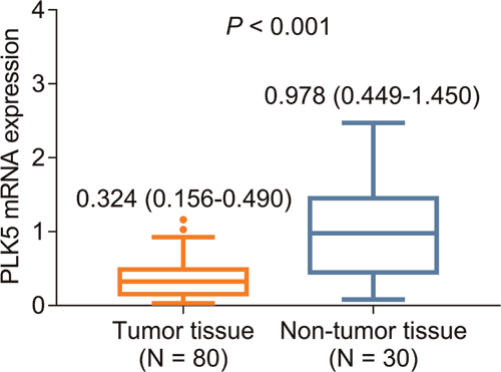
Comparison of PLK5 mRNA expression between tumor and nontumor tissues in NSCLC patients.

PLK5 mRNA expression was reduced in tumor tissue compared to nontumor tissue in NSCLC patients (*P *< 0.001). Specifically, the median (IQR) values of PLK5 mRNA expression were 0.324 (0.156–0.490) in tumor tissue and 0.978 (0.449–1.450) in nontumor tissue (Figure 3).

### Association of tumor PLK5 mRNA expression with disease characteristics

Reduced tumor PLK5 mRNA expression was correlated with increased pathological grade (*P *= 0.034), TNM stage (*P *= 0.032), and CA125 > 35 U/ml (*P *= 0.002). Nevertheless, no linkage was found between tumor PLK5 mRNA expression and other disease features, including age, gender, smoking, drinking, hypertension, hyperlipidemia, diabetes, subtype, tumor size, LYN metastasis, ECOG PS score, and CEA, in NSCLC patients (all *P *> 0.05) (Table 5).

**Table 5:** Correlation of tumor PLK5 mRNA expression with clinical characteristics.

Items	Tumor PLK5 mRNA expression, median (IQR)	*P* value
Age		0.843
≤60 years	0.321 (0.149–0.495)	
>60 years	0.326 (0.157–0.479)	
Gender		0.143
Female	0.209 (0.145–0.466)	
Male	0.364 (0.168–0.515)	
Smoking		0.769
No	0.315 (0.142–0.449)	
Yes	0.364 (0.161–0.509)	
Drinking		0.736
No	0.363 (0.149–0.495)	
Yes	0.274 (0.172–0.501)	
Hypertension		0.798
No	0.322 (0.150–0.500)	
Yes	0.324 (0.165–0.482)	
Hyperlipidemia		0.150
No	0.316 (0.148–0.455)	
Yes	0.436 (0.221–0.668)	
Diabetes		0.909
No	0.325 (0.156–0.506)	
Yes	0.311 (0.150–0.448)	
Subtype		0.602
ADC	0.308 (0.143–0.461)	
SCC	0.363 (0.172–0.539)	
Others	0.400 (0.215–0.499)	
Pathological grade		0.034
Grade 1	0.442 (0.197–0.730)	
Grade 2	0.324 (0.150–0.468)	
Grade 3	0.270 (0.142–0.430)	
Tumor size		0.821
≤5 cm	0.316 (0.190–0.449)	
>5 cm	0.346 (0.143–0.516)	
LYN metastasis		0.108
No	0.337 (0.190–0.515)	
Yes	0.219 (0.066–0.437)	
TNM stage		0.032
I	0.316 (0.205–0.422)	
II	0.392 (0.190–0.569)	
III	0.189 (0.065–0.396)	
ECOG PS score		0.868
0	0.321 (0.177–0.474)	
1	0.328 (0.103–0.563)	
CEA		0.608
≤5 ng/ml	0.316 (0.161–0.527)	
>5 ng/ml	0.328 (0.142–0.474)	
CA125		0.002
≤35 U/ml	0.400 (0.236–0.597)	
>35 U/ml	0.190 (0.129–0.408)	

PLK5, polo-like kinase 5; IQR, interquartile rage; ADC, adenocarcinoma; SCC, squamous cell carcinoma; LYN, lymph node; TNM, tumor-node-metastasis; ECOG PS, Eastern Cooperative Oncology Group performance status; CEA, carcinoembryonic antigen; CA125, cancer antigen 125.

### Correlation of tumor PLK5 mRNA expression with DFS and OS

High tumor PLK5 mRNA expression was linked with prolonged DFS in NSCLC patients (*P *= 0.046). The median (95% CI) DFS of patients with high tumor PLK5 mRNA expression was 43.0 (32.7–53.3) months; the 3-year and 5-year DFS rates were 58.1% and 29.5%, respectively. The median (95% CI) DFS of patients with low tumor PLK5 mRNA expression was 31.0 (25.7–36.3) months; the 3-year and 5-year DFS rates were 43.0% and 0.0%, respectively, in NSCLC patients (Table 6).

**Table 6:** Correlation of tumor PLK5 mRNA expression with DFS and OS.

Items	*P* value	Median (95% CI)	3-year rate	5-year rate
DFS	0.046			
High tumor PLK5 mRNA expression		43.0 (32.7-53.3), months	58.1%	29.5%
Low tumor PLK5 mRNA expression		31.0 (25.7-36.3), months	43.0%	0.0%
OS	0.107			
High tumor PLK5 mRNA expression		62.0 (not available), months	86.0%	59.1%
Low tumor PLK5 mRNA expression		49.0 (39.1-58.9), months	74.1%	35.7%

PLK5, polo-like kinase 5; DFS, disease-free survival; OS, overall survival; CI, confidence interval.

In contrast, no association was discovered between tumor PLK5 mRNA expression and OS in NSCLC patients (*P *= 0.107). In patients with high tumor PLK5 mRNA expression, the median (95% CI) OS was 62.0 (not available) months; the 3-year and 5-year OS rates were 86.0% and 59.1%, respectively. In patients with low tumor PLK5 mRNA expression, the median (95% CI) OS was 49.0 (39.1–58.9) months; the 3-year and 5-year OS rates were 74.1% and 35.7%, respectively, in NSCLC patients (Table 6).

Subsequently, data from The Human Protein Atlas (derived from TCGA analysis) were further used to verify the prognostic value of tumor PLK5 mRNA expression in NSCLC patients, and high tumor PLK5 mRNA expression was linked to longer OS (*P *= 0.046) (Supplementary Figure S1).

## Discussion

PLKs are aberrantly expressed in NSCLC patients, according to previous literature (16). A study argues that PLK1 and PLK4 are increased in NSCLC patients (16). PLK3 expression is found to be decreased in NSCLC patients (16). In terms of PLK5, studies on its expression in NSCLC are still limited. The present study found that PLK5 was decreased in tumor tissues compared to nontumor tissues in NSCLC patients. A possible explanation would be that decreased PLK5 might promote the proliferation and inhibit the apoptosis of tumor cells (20). Therefore, decreased PLK5 reflected the enhanced malignant proliferative capacity to some extent; the malignant proliferative ability of tumor tissues was more potent than that of nontumor tissues. Thus, PLK5 was decreased in tumor tissues compared with nontumor tissues in NSCLC patients.

With respect to the correlation between PLKs and clinical features in NSCLC patients, it has been demonstrated that increased PLK4 is linked with greater tumor size and LYN metastasis in NSCLC patients (18). Beyond this, both PLK1 and PLK4 positively relate to the TNM stage in NSCLC patients (16). The current study discovered that decreased PLK5 was linked to high pathological grade, the presence of LYN metastasis, increased TNM stage, and aberrant CA125 in NSCLC patients. It could be argued that reduced PLK5 would promote NSCLC cell proliferation, migration, and invasion, as well as suppress differentiation and apoptosis (20). Therefore, decreased PLK5 indicated increased pathological grade, the presence of LYN metastasis, increased TNM stage, and abnormal CA125 to a certain extent in NSCLC patients.

Simultaneously, PLKs predict the prognosis in NSCLC patients (16, 18, 21, 22). Based on previous literature, increased PLK1 is linked with a poor survival profile in NSCLC patients (21, 22). Meanwhile, high expression of PLK4 is related to shorter DFS and OS in NSCLC patients (18). Significantly, elevated PLK1, PLK4, and PLK5 levels and decreased PLK2 levels are related to unfavorable survival profiles in NSCLC patients (16). Contrary to previous findings (16), this study discovered that low PLK5 was linked with poor DFS and OS, which also independently predicted shorter DFS. (1) As mentioned above, decreased PLK5 was associated with increased pathological grade, the presence of LYN metastasis, elevated TNM stage, and aberrant CA125, while patients with these clinical features were more likely to achieve worse prognosis; thus, its low expression indicated an unfavorable prognosis. As a result, low PLK5 was related to a worse survival profile in NSCLC patients.

There were several limitations in this study: (1) this study only recruited primary NSCLC patients, and the role of PLK5 in secondary lung metastasis patients requires further exploration; (2) the linkage of PLK5 with tumor characteristics and prognosis of NSCLC patients was revealed; although some studies have reported the role of PLK5 in NSCLC progression, the specific mechanism and the relationship between PLK5 and drug resistance remain unknown; (3) this was a retrospective study, although a multivariate Cox regression model was conducted to eliminate confounding factors, some bias and compound factors might still be present; (4) the sample size could be expanded to amplify the statistical generalization; (5) this study has estimated the prognostic effect of PLK5, further study could consider exploring the relevant mechanism.

In summary, the decrease in PLK5 indicates aggravated tumor burden and an unfavorable prognosis in NSCLC patients.

## Data Availability

The original contributions presented in the study are included in the article/**Supplementary Material**, further inquiries can be directed to the corresponding author/s.
